# Study on the Vaccination of the Population of Romania against Monkeypox in Terms of Medical Security

**DOI:** 10.3390/vaccines10111834

**Published:** 2022-10-29

**Authors:** Cătălin Peptan, Vlad Dumitru Băleanu, Flavius Cristian Mărcău

**Affiliations:** 1 Faculty of Educational Sciences, Law and Public Administration, “Constantin Brâncuși” University of Târgu Jiu, 210185 Târgu Jiu, Romania; 2 Faculty of Medical and Behavioural Sciences, “Constantin Brâncuși” University of Târgu Jiu, 210185 Târgu Jiu, Romania

**Keywords:** monkeypox, vaccine, hesitation, acceptance, human safety, romania

## Abstract

Although it has been shown in numerous studies that immunization of the population by vaccination is the most effective way to protect against smallpox or other polioviruses, the anti-vaccination public rhetoric recorded during the COVID-19 pandemic is likely to influence the populations acceptance of vaccination against newly emerging viruses. This fact influenced our decision to study the vaccination of the Romanian population against the virus that causes monkeypox, aiming to identify the degree of compliance regarding the decision related to vaccination acceptance/non-acceptance/hesitation, based on the survey of a representative sample of respondents. The study is based on an online questionnaire completed between 1 July and 31 July 2022 by 820 individuals, aged 18 years or above, with a permanent residency in Romania. The study was undertaken in order to observe the attitudes of the respondents regarding the acceptance, refusal, or hesitation of vaccination against monkeypox. The sociological data resulting from the application of the questionnaire on 820 people highlighted that 97.16% were vaccinated with the vaccines of the national mandatory scheme and 53.32% were vaccinated with the optional vaccines (rotavirus vaccine, anti-hepatitis A, meningococcal vaccine, etc.). Although 47.13% of respondents considered monkeypox to be a real problem facing humanity today, only 26.37% of those surveyed expressed their fear of becoming infected, and 29.30% were willing to immunize themselves against the virus by vaccination. Only 19.59% of respondents believed that the monkeypox disease will generate a new global pandemic, while 31.86% considered pandemics to be a human security issue, and 30.28% expressed their desire to accept a reduction in some rights and freedoms, in the short term, for the adoption of institutional measures to combat a possible pandemic caused by monkeypox. The study clearly highlights the fact that monkeypox is perceived as a threat to the health of the population, with relatively low acceptance of conspiracy theories regarding its origins/manifestation/consequences among respondents (between 21.7% and 28.9%). The vaccination of the population against monkeypox is strongly influenced by the validity of the results obtained over time, in the vaccination campaigns against the smallpox virus (vaccine found in the mandatory vaccination scheme in Romania until 1979). We believe that the negative public rhetoric regarding the COVID-19 vaccination is likely to negatively influence monkeypox vaccination. Although specialized studies and practical results showed that the immunization of the population through vaccination represents an important vector in the prevention/management of pandemic-type issues, we believe that a national pro-vaccination campaign, based on scientific evidence, can lead the population to accept vaccination when the epidemiological context requires it. We also believe that a culture of health security needs to be developed among citizens to raise awareness of the role of vaccines as an important vector in the field of population health.

## 1. Introduction

Monkeypox (MPX) is a rare viral infection caused by a virus classified as a poxvirus. The virus is transmitted directly or indirectly from animals to humans, but the spread of the virus from person to person is possible through large respiratory droplets, direct contact and contaminated fomites [1,2]. Singhal, Kabra, and Lodha show that the natural reservoirs are monkeys, squirrels, Gambian pouched rats, dormice, nonhuman primates, and other species, and that the disease can also be contracted as a result of scratching, biting, and eating improperly cooked meat from infected animals [3]. Titanji et al. believe that the natural animal reservoir was not fully identified, as some variables are not yet known, such as the lack of information on the asymptomatic course of the disease in animals [4].

Monkeypox is a double-stranded DNA virus belonging to the genus Orthopox in the Poxviridae family, and its closest known relatives are the Variola virus, the causative agent of smallpox, and the Vaccinia virus (VACV) [5]. In terms of comparative genomic analysis between monkeypox and smallpox, it is observed that the former is a discrete species with multiple differences in virulence genes from major and minor strains of smallpox [6]. It is considered that the two probably evolved independently from an Orthopox virus ancestor similar to Cowpox [6].

Smallpox is believed to have transgressed into humans approximately four thousand years ago, and the first possible vaccine design appeared in 1796 with Jenner’s cowpox vaccine. Vaccination made it possible to eradicate smallpox in 1980 [7].

The incubation period of monkeypox is generally between four and 14 days, with symptoms characterized by vesicular rashes or lesions on the body surface, fever, sore throat, enlarged lymph nodes, lesions in the mouth and eyes, and acute muscle pain [8,9].

The first case of human infection was recorded in 1970 in the Democratic Republic of Congo, with Central and West Africa subsequently representing the geographical area of the most cases of infection with of respective virus globally [10].

More recently, in 2003, human cases of monkeypox were identified by the Centers for Disease Control and Prevention in the United States of America. The likely source of the virus was the import of live animals from Ghana, but no deaths were recorded [10].

The year 2022 is characterized by the manifestation of monkeypox cases all over the world, including European Union states such as Austria, Belgium, France, Germany, Italy, Portugal, Spain, Sweden, Romania and the Netherlands, etc. [11]. Investigations reported that not all diagnosed individuals had travelled to regions where the disease is endemic, an aspect that makes it difficult to accurately establish the source of infection. Epidemiological evaluations carried out by the European Center for Disease Prevention and Control (ECDC) show a high degree in the spread of the disease in people who have several sexual partners [11], but also in children or immunosuppressed people [12].

On 15 August 2022, there were over 15,659 confirmed cases of monkeypox in the European Union, most of them in Spain (5719), Germany (3142), the United Kingdom (3081), France (2673), the Netherlands (1029), Portugal (770), and Italy (644) [13]. As a result, the European Commissioner for Health, Stella Kyriakides, expressed concern regarding the “increasing number of cases of monkeypox in the European Union” [14]. The United Kingdom of Great Britain and Northern Ireland recorded a number of 3081 confirmed cases on the previously mentioned date [15].

In Romania, the first case of monkeypox infection was identified on 13 June 2022, and as of 15 August 2022, 32 infected persons were identified, all of them male, aged between 22 and 51 years old [16]. No serious cases affecting the health of those infected were recorded.

The increasing European case rate encouraged the Committee for Medicinal Products for Human Use (CHMP) of the European Medicines Agency (EMA) to assess the appropriateness of expanding the use of the Imvanex vaccine against smallpox and monkeypox, due to the similarity between the viruses at their origin [17]. The prompt reaction of the European Commissioner for Health to counter the spread of the virus at a continental level, especially in areas with major potential for spreading, led to the allocation of approximately 25,000 doses of vaccine to the affected states, as follows: Spain, Germany, and Italy received 5300 doses, Belgium received 3040 doses, Sweden and Portugal received 2700 doses, and Ireland received 1400 doses. Norway and Iceland will be allocated the necessary doses in the near future. It should also be mentioned that the European Commission purchased 163,620 doses of vaccine to combat the spread of monkeypox in 20 July 2022 [14]. Due to the accelerated rate of virus spread on the European continent, as well as the moderate global expansion, the World Health Organization (WHO) declared the outbreak of monkeypox as a global health emergency, calling for the acceleration of research for the production of effective vaccines and the implementation of measures to limit the spread of the virus [18]. In terms of vaccinating the population with a smallpox vaccine, it was proven that vaccinated individuals were much better protected against monkeypox by developing less severe forms of the disease compared with those who did not receive a vaccine. Furthermore, if the vaccine is administered within four days of infection, it can modify or prevent the onset of clinical disease [6,7].

It should be noted that the spread of the monkeypox virus in Europe occurred more than two years after the WHO declared the COVID-19 pandemic, caused by the spread of SARS-CoV-2 (Severe Acute Respiratory Syndrome Coronavirus 2), a pandemic considered to be a real threat to human security, both from a medical perspective and in terms of related effects [19,20]. In the context of the ongoing and unpredictable dynamics of the COVID-19 pandemic and the negative rhetoric regarding the effects of vaccination against COVID-19, the willingness of the population to accept vaccination against the monkeypox virus can be decisively influenced. Against this background, it is worth noting the position of the Director General of the WHO, Dr. Tedros Adhanom Ghebreyesus, at the Brussels World Summit on Vaccination in September 2019. He campaigned for combating the spread of diseases through the immunization conferred by vaccination (this problem being considered “a global challenge” by the President of the European Commission, Jean-Claude Juncker [21]), claiming vaccination could contribute substantially to the management of some subsequent issues of human security, viewed from a medical perspective [2].

Our study is focused on determining the degree of acceptance or refusal of the respondents regarding monkeypox vaccination.

The purpose of the research is to obtain sufficient data to allow us to extract viable answers to various questions, according to Table 1, in order to determine the cause–effect relationship for acceptance/rejection of the monkeypox vaccination.

## 2. Materials and Research Methods

### 2.1. Participants

The study was developed by processing the data obtained through an online questionnaire between 1 July 2022–31 July 2022. The minimum conditions imposed for a person to be included in the interviewed target group were the following: (1) minimum age 18 and (2) permanent residence in Romania.

Participation was voluntary, with no coercive elements. Before completing the questionnaire, the respondents were informed about the authors of the study, their affiliation, the purpose of the study, and the source of funding for the research.

### 2.2. Procedure

The applied questionnaire was elaborated on the Google Forms platform and distributed through the associated link on social media platforms, aiming to cover all geographical regions of Romania. No respondent identification data were requested. Completion was only possible for people who were at least 18 years old.

### 2.3. Measurements

Socio-demographic data (age, residence, education, county of residence), data regarding the respondents’ opinions on the administration of the vaccines included in the mandatory vaccination scheme in Romania and of the optional vaccines, and data on confidence in monkeypox vaccination were collected. This data allowed us to: (a) show the behavioral attitude of the respondents regarding the acceptance or non-acceptance of vaccination against monkeypox; (b) show the behavior of the participants towards “fake news” claims related to the monkeypox vaccination process; and (c) show respondents’ opinion towards classifying pandemics as threats to human security (from a medical perspective) and the degree of acceptability of diminishing certain rights and freedoms through institutional measures to combat the effects of a potential pandemic.

### 2.4. Statistical Data Analysis

The analysis and processing of the questionnaire data was carried out using the Excel program, part of the Microsoft Office Professional Plus 2019 package, installed on a computer running Microsoft Windows 11 Professional.

The variables that were the basis for the analysis of the data concerning the respondents were: (1) age range; (2) level of graduate education; (3) residence environment (urban/rural); and (4) acceptance/non-acceptance of mandatory, optional (rotavirus vaccine, anti-hepatitis A, meningococcal vaccine, etc.), and anti-COVID-19 vaccines. Following the statistical analysis, we proceeded to compare the results to observe the respondents’ behavior according to the 4 previously mentioned variables. The answers given in the second part of the questionnaire show the level of trust given in the information found in “fake news”, the opinion regarding the framing of pandemics in the category of threats to human security, and the degree of acceptability of diminishing individual rights and freedoms to combat the effects of a potential pandemic.

The questionnaire allowed us to extract a set of data, which we analyzed statistically to extract percentages, distribution frequency, medians, and standard deviation. In order to determine the degree of correlations between certain selected variables, we used Kendell and Spearman statistical tests. Thus, we were able to observe if there was any significant degree of correlation between different variables and the participants’ decision to vaccinate/not vaccinate against monkeypox.

## 3. Results

The conducted study is based on the analysis of 820 valid answers, given through the applied questionnaire. The socio-demographic data of the participants are presented in Table 2.

### 3.1. Analysis of the Level of Acceptance of Vaccination as a Way to Prevent Viral Infections

The analysis of the data collected on vaccination with the vaccines included in the Romanian mandatory national scheme with the optional vaccines (rotavirus vaccine, anti-hepatitis A, meningococcal vaccine, etc.), respectively, reveals a very high percentage of respondents who were vaccinated with the vaccines in the first category (97.1%) and a significantly lower percentage (53.3%) of respondents vaccinated with the vaccines in the second category. Furthermore, 69.3% of respondents declared that they were vaccinated against COVID-19 (Table 3).

With respect to the two categories of vaccines, the comparative analysis of the degree of confidence among them reveals a level of confidence of 89.4% in the vaccines included in the national mandatory scheme in Romania, respectively, and 74.3% in the optional vaccines, other than COVID-19 (Table 4). It is observed that the difference in the degree of confidence of mandatory vaccines and optional vaccines is 15%.

### 3.2. Analysis of Monkeypox, the New Global Medical Emergency

In the context of the global appearance of monkeypox, a percentage of 89.1% of respondents (731 people) declared that they were aware of this issue, while 78.6% (645 people) were aware that cases of monkeypox had been found in Romania. At the same time, 26.3% of respondents expressed their fear of being infected with monkeypox, and 29.3% expressed their agreement to be vaccinated against monkeypox (Table 5). It is noteworthy that 19.5% of respondents believe that monkeypox will become a new pandemic that will manifest itself globally (Table 5).

### 3.3. Analysis of Reasons for Acceptance/Non-Acceptance of Monkeypox Vaccine Administration

Regarding the answers provided by the respondents to the open question, “what makes you decide to vaccinate/not to vaccinate against monkeypox”, we note the following arguments in Table 6 and Table 7. Pro (N = 251/30.6%) and against (N = 569/69.3%) vaccination:

### 3.4. Analysis of Participants’ Confidence in Monkeypox Information

In the second section of the questionnaire, the aim was to analyze the respondents’ behavior as a result of the emergence of information on monkeypox in the public area (origin, manifestation, evolution, consequences, etc.). The allegations presented in Table 8 were selected as a result of fake news information emerging in the public area. As in the case of COVID-19 [22,23,24,25,26,27,28,29] and in the monkeypox situation, conspiracy theories are circulating on the internet in Romania. In the case of the SARS-CoV-2 vaccination campaign, such conspiratorial information led to a low rate of acceptance of vaccination in Romania [22,23]. Following the completion of the questionnaire, the obtained results are presented in Table 8.

## 4. Discussion

Regarding willingness to accept the vaccines included in the national scheme, it is noted that the number of people surveyed who were vaccinated with these vaccines is considerably higher than the number who were vaccinated with optional vaccines other than COVID-19 (97.1% compared to 53.3% of all respondents). The highest percentage of people vaccinated with mandatory vaccines is found in the 56–60 and 66+ age categories (100%), followed by the 36–40 age category (97.7%). In the case of optional vaccines, we find a peak in the age category of 46–50 (71.8%), with the lowest percentage being found in the age categories 66+ (40.7%) and 26–30 (48.8%).

The highest percentages of participants’ confidence in the mandatory vaccines included in the national scheme are found in the age categories 56–60 (97.1%) and 46–50 (95.3%), and in the case of optional vaccines in the categories aged 18–20 (86.3%) and 56–60 (82.8%). Per a contrario, the lowest percentage of confidence in the case of mandatory vaccines is found in the age categories 61–65 (70%) and 66+ (85.1%), and for optional vaccines in the age categories 61–65 (56.6%) and 26–30 (65.4%).

The anti-COVID-19 vaccines enjoyed a fairly high level of confidence, with 70.1% of respondents declaring that they are vaccinated with one of the vaccines available in Romania. The highest percentage of vaccination is found in the 51–55 age group (80.3%), and the lowest in the 66+ category (44.4%), although this age group is the most affected by SARS-CoV-2 virus.

According to the previously presented information, it can be concluded that there is a willingness of the participants to be vaccinated, both with the vaccines included in the national scheme and with the optional vaccines [22], including those against COVID-19 [23]. However, in the case of a potential vaccination against monkeypox, we find a reluctance among the respondents, where only 29.3% would agree to be vaccinated against this constantly expanding disease, although the fear of the new disease is found in 26.3% of the surveyed respondents (Table 5).

The reasons indicated by the respondents for accepting vaccination against monkeypox (Table 6) are mainly based on their desire to be safe, protected against the disease (22.3%), preservation of their own health (21.9%), and trust in vaccines and science (17.5%). In the case of participants who chose not to vaccinate (Table 7), the main reasons are mistrust in the vaccine (18.6%), possible side effects of the vaccine (12.8%), low degree of contagion of the disease (8.6%), and disbelief in the existence of the disease (8%). We are of the opinion that such reasons are based, as in the case of COVID-19 vaccines, on “fake news” information that is found in the public space, as it is well known that Romania had one of the lowest vaccination rates against the SARS-CoV-2 virus [22,23,25,26,27,28,29].

The fear of the disease as a determining reason for accepting/rejecting vaccination is represented in Table 6.

**Figure 1:**
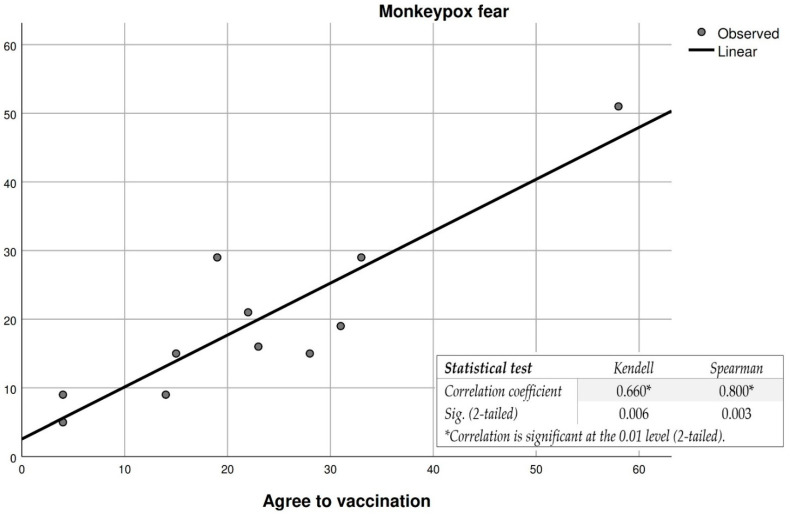
Correlation between fear of the monkeypox disease and the participants’ decision to vaccinate against the disease. * For a correlation to be very strong, the correlation coefficient must be as close as possible to 1, and sig. as close as possible to 0.

**Figure 2:**
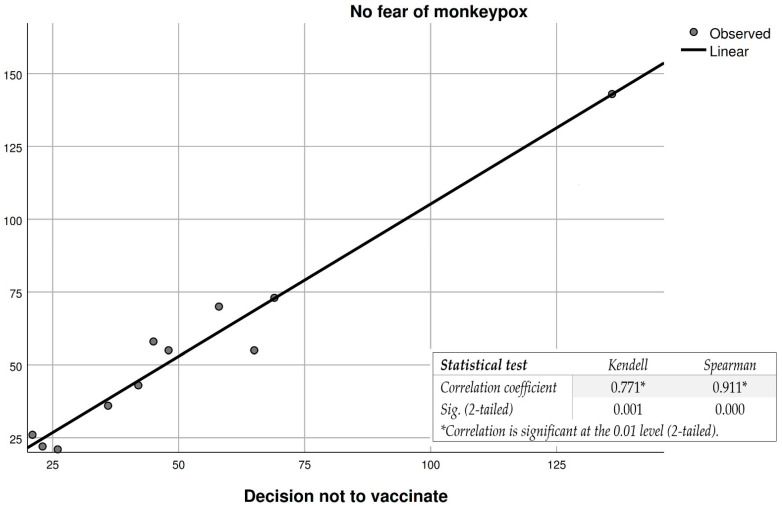
Correlation between fear of the monkeypox disease and the participants’ decision not to vaccinate against the disease. * For a correlation to be very strong, the correlation coefficient must be as close as possible to 1, and sig. as close as possible to 0.

The variable “people’s fear of getting sick” correlated with participants’ decision to get vaccinated (Figure 1 and Figure 2), and reveals a moderate to good correlation in the case of the Kendell and Spearman tests. However, in the case of participants with no fear of the disease and their decision not to vaccinate, the correlation is significantly stronger for both tests. This leads to the conclusion that the variable “fear of getting sick” is not a decisive constant in the participants’ intention to vaccinate against monkeypox, which can also be seen in the list of reasons for participants (11.1%) choosing to vaccinate (Table 6). Overall, from the application of statistical tests, we understand that people with an increased fear of monkeypox are likely to accept the inoculation of a vaccine that will prevent them from getting sick or, in the case of a potential infection, the symptoms to be mild. In contrast, participants who said they were not afraid of getting sick refused vaccination against the disease. Such results were also observed in studies that addressed vaccine acceptance for COVID-19 [25,26,27,28,29].

**Figure 3:**
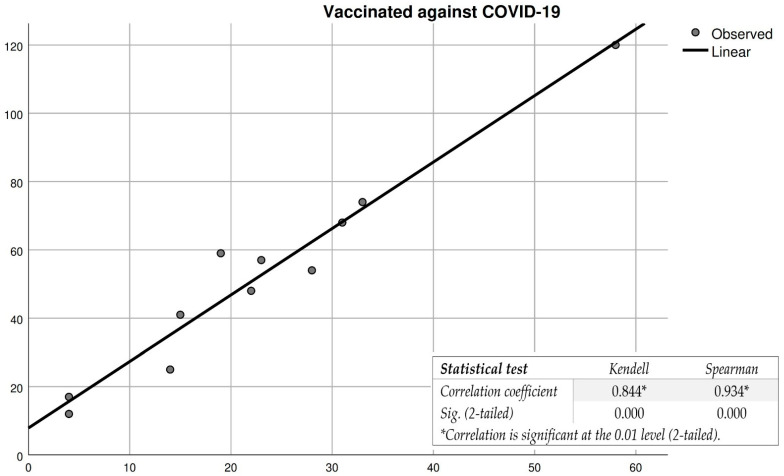
Correlation between participants’ vaccination against COVID-19 and their decision to vaccinate against monkeypox. * For a correlation to be very strong, the correlation coefficient must be as close as possible to 1, and sig. as close as possible to 0.

Regarding the determination of the correlation between people who chose to vaccinate against COVID-19 and those who accept vaccination against monkeypox, Figure 3 shows that both the Kendell and Spearman correlation indices show a very good degree of association (correlation is strong). The COVID-19 vaccination indicator of individuals can be taken into account in the case of large-scale vaccination against monkeypox. Participants vaccinated against COVID-19 show a high degree of acceptance of vaccination against monkeypox as a result of their confidence in the vaccines.

**Figure 4:**
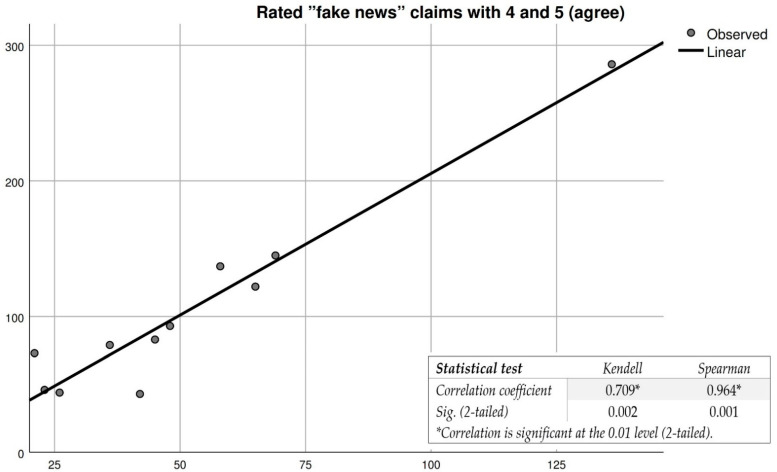
Correlation between participants’ trust in “fake news” information and their decision not to vaccinate against monkeypox. * For a correlation to be very strong, the correlation coefficient must be as close as possible to 1, and sig. as close as possible to 0.

With regard to the second part of the questionnaire (Table 8), it should be noted that in question Q1, 47.1% of the respondents agree that monkeypox is a real problem in today’s society. For questions Q2–Q6 (which can be classified as “fake news” information regarding the monkeypox disease), 55.8% and 64.3% of the respondents disagree and 21.7% and 28.9% out of the respondents agree with the allegations, respectively. Correlation tests also show a clear link between the decision to vaccinate and confidence in “fake news” claims, as shown in Figure 4. In this situation, we observed that participants who place a high degree of trust in conspiratorial “fake news” information have an extremely low degree of acceptance of the monkeypox vaccine inoculation. Such situations are also observable in studies dealing with the acceptance of COVID-19 vaccines [22,23].

The public dissemination of “fake news” information and the participants’ trust in such information are likely to lead to the adoption of the decision not to vaccinate against monkeypox.

Regarding questions Q7–Q8 (which classify sexual intercourse as the origin of the monkeypox disease), 53.9% and 71.6% of respondents disagree, while 11.6% and 21.4% of respondents agree with the veracity of the subject matter, respectively.

Regarding questions Q9–Q10 which consider the categorization of pandemics as threats to human security and the willingness of respondents to agree to the adoption of institutional measures to combat a possible pandemic caused by monkeypox, 31.86% and 30.28% of respondents agree on the subject, respectively.

## 5. Research Limitations

In addition to the strong points of view of this study, it is also one of the first studies in Romania dealing with the management of monkeypox at the national level, and therefore it has also some limitations.

The first important limitation, which could have revealed a much higher degree of rejection of vaccination, is given by the low percentage of respondents with pre-university education (22.6%) compared to the rest of the respondents. In this case, this category of individuals shows a much greater vaccine hesitation or rejection of the vaccine in general [22,23].

The second limitation is due to the fact that the present research was conducted during the sixth wave of the COVID-19 pandemic, against the background of negative public rhetoric related to the effectiveness of the COVID-19 vaccination. In this context, it is possible that respondents were influenced by this state of affairs and refused to vaccinate against monkeypox.

The present research is strictly related to the information obtained from the questionnaire, and therefore the third limitation is the fact that the distribution of the questionnaire was undertaken online, with the possibility of subjective self-selection of the participants [30]. Furthermore, only participants who had an internet connection were able to answer the questions, and many people over 60 years old do not use the internet and social media platforms [31]. In addition, the questionnaire could be redistributed, with the possibility of exaggerating a particular point of view, as the respondents might share the questionnaire with people they know have similar interests and perspectives [31].

## 6. Conclusions

The study highlights the fact that 47.1% of the respondents (487 people) consider monkeypox as a real threat to public health. A total of 89.1% of the respondents (731 people) declared that they are aware of the existence of the disease at the global level, while 78.6% (645 people) knew that cases of infected people had also been detected in Romania.

On the other hand, the study reveals that the degree of acceptance of conspiracy theories regarding the origins/manifestation/consequences of monkeypox is relatively low among the respondents, ranging from 21.7% to 28.9%. This fact is likely to create favorable prospects for the adoption of institutional measures in order to prevent the spread of the virus in Romania.

In terms of institutional action to prevent the spread of monkeypox, it is concluded that vaccination of the population is strongly influenced by the validity of the results obtained over time in smallpox vaccination campaigns (vaccine found in the mandatory vaccination scheme in Romania until 1979 [24]) and against COVID-19. We believe that the negative public rhetoric regarding COVID-19 vaccination is likely to negatively influence vaccination in general [24,25,26,27,28,29] and the monkeypox vaccination in particular [32].

Whereas specialized studies and practical results have showed that immunization of the population through vaccination represents an important vector in the prevention/management of issues such as pandemics [33], we believe that a national pro-vaccination campaign, based on scientific evidence, can persuade the population to accept vaccination against monkeypox, if the epidemiological situation so requires.

We also believe that it is necessary, through concerted actions of state institutions and civil society, to develop the culture of medical security among citizens to make them aware of the role of vaccines as an important vector of critical infrastructure in the field of population health. This approach is also motivated by some of the results of this study, which highlight the fact that only 31.8% agreed on the inclusion of pandemics in the category of threats to human security, and 30.2% of respondents expressed their degree of readiness to accept the adoption of institutional measures to combat a possible monkeypox pandemic.

## Figures and Tables

**Table 1:** Data required and research questions.

Required Data	Questions
Determining participants’ fears regarding monkeypox disease	Does participants’ fear of getting sick lead them to accept vaccination?
The degree of acceptance of the participants for the anti-COVID-19 vaccination	Will people vaccinated with COVID-19 vaccines accept the monkeypox vaccination?
Determining the degree of trust in “fake news” information	Will people who believe fake news reject the monkeypox vaccination?

**Table 2:** Socio-demographic data of the participants.

Age	Sex					Environment of Residence		Educational Level				
	Females		Males		N. A.	Urban	Rural	Less than High School	High School or Equivalent	Bachelor’s Degree	Master’s Degree	Doctoral Degree
	N	%	N	%	N							
18–20	31	3.7	42	5.1	0	41	32	1	27	45	0	0
21–25	98	11.9	92	11.2	4	116	78	0	39	124	31	0
26–30	44	5.3	38	4.6	2	57	27	0	13	41	28	2
31–35	56	6.8	44	5.3	2	76	26	1	18	37	42	4
36–40	47	5.7	41	5	1	73	16	2	20	33	27	7
41–45	37	4.5	34	4.1	0	51	20	0	17	28	21	5
46–50	32	3.9	32	3.9		50	14	0	9	27	22	6
51–55	23	2.8	28	3.4	0	41	10	0	4	24	14	9
56–60	18	2.2	16	1.9	1	22	13	0	15	9	8	3
61–65	14	1.7	15	1.8	1	19	11	6	6	15	3	0
66+	12	1.4	14	1.7	1	19	8	3	5	12	4	3

N—number; N. A.—not answering.

**Table 3:** The degree of vaccination of participants with mandatory, optional, and anti-COVID-19 vaccines.

Age Range	Were You Vaccinated as a Child with the Mandatory Vaccines?	Were You Vaccinated with the Optional Vaccines?	Were You Vaccinated against COVID-19?
	Yes %	Yes %	Yes %
18–20	97.2	61.4	73.9
21–25	96.3	55.6	61.8
26–30	96.4	48.8	70.2
31–35	99	52.9	72.5
36–40	97.7	61.8	76.4
41–45	97.1	66.2	80.2
46–50	95.3	71.8	75
51–55	96.0	70.5	80.3
56–60	100	54.2	71.4
61–65	93.3	63.3	56.6
66+	100	40.7	44.4
Descriptive statistics			
Mean	0.97	0.05	0.69
Standard Error	0.00	0.00	0.03
Median	0.97	0.05	0.72
Standard Deviation	0.02	0.03	0.10
Sample Variance	0.00	0.00	0.01
Kurtosis	0.01	3.49	1.57
Skewness	−0.18	1.41	−1.37
Confidence Level (95.0%)	0.01	0.02	0.07

**Table 4:** Participants’ confidence in vaccines included in the national vaccination scheme and optional vaccines.

Age Range	Confidence in the Mandatory Vaccines Administered during Childhood		Confidence in Vaccines That Are Optionally Administered, Other Than Anti-COVID-19	
	Yes %		Yes %	
18–20	91.7		86.3	
21–25	86		77.3	
26–30	89.2		65.4	
31–35	90.2		75.4	
36–40	91		75.2	
41–45	94.3		80.2	
46–50	95.3		81.2	
51–55	94.1		70.5	
56–60	97.1		82.8	
61–65	70		56.6	
66+	85.1		66.6	
Descriptive statistics	Mean	0.89	Mean	0.74
	Standard Error	0.02	Standard Error	0.02
	Median	0.91	Median	0.75
	Standard Deviation	0.07	Standard Deviation	0.08
	Sample Variance	0	Sample Variance	0
	Kurtosis	4.77	Kurtosis	0.04
	Skewness	−1.96	Skewness	−0.7
	Confidence Level (95.0%)	0.05	Confidence Level (95.0%)	0.05

**Table 5:** Participants’ opinion on monkeypox.

Age Range	Do You Think Monkeypox Will Turn into a Pandemic?	Are You Afraid That You Will Get Monkeypox?	Would You Agree to Get Vaccinated against Monkeypox?
	Yes %	Yes %	Yes %
18–20	26	20.5	38.3
21–25	34.5	26.2	29.9
26–30	30.9	34.5	22.6
31–35	22.5	28.4	32.3
36–40	16.8	21.3	34.8
41–45	16.9	22.5	32.3
46–50	12.5	32.8	34.3
51–55	5.8	29.4	29.4
56–60	17.1	25.7	40
61–65	10	30	13.3
66+	22.2	18.5	14.8
Descriptive statistics			
Mean	0.19	0.26	0.29
Standard Error	0.02	0.01	0.02
Median	0.17	0.26	0.32
Standard Deviation	0.087	0.05	0.08
Sample Variance	0.00	0.00	0.00
Kurtosis	−0.52	−1.10	−0.18
Skewness	0.22	0.02	−0.90
Confidence Level (95.0%)	0.05	0.03	0.05

**Table 6:** Arguments of participants who accept vaccination against monkeypox.

Arguments for Vaccination	N = 251	%
To be safe/to be protected against disease	56	22.3
For your own health	55	21.9
Vaccine trust and science	44	17.5
Fear of monkeypox	28	11.1
Not to pass the disease to other people	22	8.7
Trust in the medical system and doctors	16	6.3
Other reasons	20	7.9

**Table 7:** Arguments of participants who do not accept vaccination against monkeypox.

Anti-Vaccination Arguments	N = 569	%
Mistrust in the vaccine	106	18.6
Possible side effects of the vaccine	73	12.8
Reduced transmission of the disease	49	8.6
The disease does not exist	46	8
Insufficiently tested vaccine	44	7.7
The vaccine is an experiment on humans	33	5.7
Mistrust in vaccines as a result of the COVID-19 pandemic	25	4.3
Lack of information from officials	24	4.2
Few studies on the disease	16	2.8
Other reasons or lack of response	152	26.8

**Table 8:** Rating of “fake news” statements by survey participants.

	Fake News Allegations	Disagreement		Uncertain		Agreement	
		N	%	N	%	N	%
Q1	Is monkeypox real?	257	31.3	176	21.4	386	47.1
Q2	Was there a worldwide occult that spread monkeypox which wants to depopulate the earth?	494	60.3	135	18.4	190	23.2
Q3	Did monkeypox emerge in the wake of the COVID-19 pandemic to keep the world’s population in check through fear?	457	55.8	125	15.2	237	28.9
Q4	Are smallpox vaccines made to reduce the earth’s population, just like the talk of COVID-19 vaccines?	518	63.2	118	14.4	183	22.3
Q5	Are doctors paid to induce panic among the population?	527	64.3	114	13.9	178	21.7
Q6	Was monkeypox created because the population was not sufficiently reduced as a result of the COVID-19 pandemic?	520	63.4	115	14.0	184	22.4
Q7	Did monkeypox emerge in the aftermath of the COVID-19 pandemic to keep the world’s population under control through fear?	485	59.2	119	14.5	215	26.2
Q7	Do you think it is true that unprotected sexual contact is at the origin of the spread of the monkeypox disease?	442	53.9	201	24.5	176	21.4
Q8	Do you think the statement which claims that the propagation of the monkeypox disease lies in unprotected sexual contact has the purpose of reducing the population of the planet?	587	71.6	137	16.7	95	11.6
Q9	Do you agree with the authorities’ point of view that pandemics are limited to human security issues?	342	41.7	216	26.3	261	31.8
Q10	Would you agree to accept the curtailment of some freedoms, in the short term, in order to take institutional measures to combat a possible monkeypox pandemic?	441	53.8	130	15.8	248	30.2

## Data Availability

Data can be requested from the corresponding author.
